# Cross-Cultural Adaptation and Validation of the Kazakh Version of the WOMAC Index in Patients with Knee Osteoarthritis

**DOI:** 10.3390/ijerph23040445

**Published:** 2026-03-31

**Authors:** Yerden Khaumet, Almasbek Akhmetov, Ikilas Moldaliyev, Azamat Seksenbayev, Ainash Oshibayeva, Saltanat Kyrykbayeva, Gulnaz Nuskabayeva, Akylbek Ibragim

**Affiliations:** 1 Department of Traumatology and Orthopedics, Clinical and Diagnostic Center, Akhmet Yassawi International Kazakh-Turkish University, Turkestan 161200, Kazakhstan; yerden.khaumet@ayu.edu.kz (Y.K.); ortho.akylbek@gmail.com (A.I.); 2 Faculty of Medicine, Akhmet Yassawi International Kazakh-Turkish University, Turkestan 161200, Kazakhstan; moldaliev@ayu.edu.kz (I.M.); ainash.oshibayeva@ayu.edu.kz (A.O.); saltanat.kyrykbayeva@ayu.edu.kz (S.K.); nuskabayeva.gulnaz@ayu.edu.kz (G.N.); 3 Independent Researcher, Astana 010000, Kazakhstan; seksenbayev@gmail.com

**Keywords:** knee osteoarthritis, WOMAC, validation

## Abstract

**Highlights:**

**Public health relevance—How does this work relate to a public health issue?**

- Knee osteoarthritis is a substantial contributor to disability, requiring reliable patient-reported outcome measures across languages.
- This study presents the first validated Kazakh version of the WOMAC index.

**Public health significance—Why is this work of significance to public health?**

- Enables accurate assessment of pain and functional limitation in Kazakh-speaking populations.
- Supports improved monitoring of osteoarthritis burden in Central Asia.

**Public health implications—What are the key implications or messages for practitioners, policy makers and/or researchers in public health?**

- Facilitates routine clinical assessment and outcome evaluation in knee osteoarthritis.
- Strengthens epidemiological research and evidence-based public health planning.

**Abstract:**

Knee osteoarthritis (KOA) is a prevalent condition associated with pain and reduced physical function worldwide, and the Western Ontario and McMaster Universities Osteoarthritis Index (WOMAC) is one of the most commonly used disease-specific patient-reported outcome measures. Its use in non-English-speaking populations requires appropriate translation and validation, and no validated Kazakh version has previously been available. This study aimed to translate, culturally adapt, and evaluate the psychometric properties of the Kazakh version of the WOMAC in patients with KOA. A cross-sectional validation study was conducted among 452 patients with clinically diagnosed KOA and 126 healthy individuals, following established international guidelines. The study assessed internal consistency, test–retest reliability, construct validity, content validity, convergent validity, known-groups validity, and floor and ceiling effects. The Kazakh WOMAC demonstrated acceptable to high internal consistency (Cronbach’s α = 0.77–0.88) and good test–retest reliability (ICC = 0.78–0.83). Content validity was excellent (S-CVI/Ave = 0.96), and confirmatory factor analysis supported the original three-factor structure. Expected correlations with SF-36 domains confirmed convergent validity, and WOMAC scores differentiated patients with KOA from healthy individuals, with no relevant floor or ceiling effects observed. The Kazakh version of the WOMAC is a reliable and valid instrument for assessing pain, stiffness, and physical function in Kazakh-speaking patients with KOA.

## 1. Introduction

Osteoarthritis (OA) is one of the most prevalent chronic musculoskeletal disorders globally and a leading cause of pain, disability and reduced quality of life [1,2]. Estimates based on the Global Burden of Disease (GBD) 2021 report indicate that approximately 607 million individuals experienced OA in 2021, with knee osteoarthritis (KOA) representing the most prevalent and disabling form of the disease [3]. The burden of OA continues to rise with global demographic shifts, including population aging and increases in high body mass index, which exacerbate the incidence and impact of the disease [1,2,3].

Patient-reported outcome measures are essential for capturing the patient perspective on pain, stiffness and functional limitations [4]. Among disease-specific instruments, the Western Ontario and McMaster Universities Osteoarthritis Index (WOMAC) remains one of the most widely used tools in both knee and hip osteoarthritis research [5,6]. The WOMAC comprises three subscales assessing pain, stiffness and physical function, and is widely applied in clinical trials and epidemiological studies to assess disease impact and intervention efficacy. It is also widely used in routine clinical practice as a primary or secondary outcome measure [4,7].

The measurement properties of the WOMAC have been investigated in multiple populations and settings. Contemporary studies continue to demonstrate acceptable internal consistency, test–retest reliability and construct validity across different groups and modes of administration [5,7]. Recent research confirms that both the full and abbreviated versions of the WOMAC maintain adequate psychometric performance in patients with KOA, supporting its continued relevance as an outcome measure [5]. Furthermore, studies testing the equivalence of paper-based and electronic versions have reinforced its utility in modern clinical and research environments [8].

As the WOMAC was originally created in English, its implementation in other languages requires careful translation and culturally appropriate adaptation [9]. Linguistic differences, cultural practices and variations in daily activities may influence how patients interpret questionnaire items and report symptoms [9,10]. Without appropriate adaptation and validation, the validity and reliability of these measures may be compromised when applied in new cultural contexts [9,10,11]. International guidelines emphasize the importance of systematic translation procedures and psychometric evaluation to ensure conceptual and measurement equivalence [9].

Validated translations of the WOMAC have been produced in numerous languages including Korean, Chinese, Turkish, Arabic and Moroccan Arabic, with studies consistently demonstrating acceptable reliability and validity in patients with KOA [11,12,13,14,15]. These findings underscore the importance of evaluating each language version independently rather than assuming equivalence across populations [11,12,13,14,15].

Despite the growing burden of osteoarthritis in Central Asia, no validated Kazakh language version of the WOMAC is currently available [1,2,3]. The absence of a culturally adapted and psychometrically tested instrument limits accurate patient-centered assessment and constrains research on KOA in Central Asia, where culturally specific behaviors and environmental factors play an important role in disease risk and progression [9,16].

This study aimed to translate, culturally adapt, and examine the reliability and validity of the Kazakh version of the Western Ontario and McMaster Universities Osteoarthritis Index (WOMAC), using the full 24-item Likert-type version (LK 3.1), among patients with KOA.

## 2. Materials and Methods

### 2.1. Study Design and Participants

This study employed a cross-sectional design to translate, culturally adapt, and evaluate the psychometric properties of the Kazakh version of WOMAC LK 3.1. Participants were recruited using a convenience sampling strategy from the outpatient Department of Traumatology and Orthopedics at the Clinical and Diagnostic Center of Akhmet Yassawi International Kazakh–Turkish University. Patients presenting for routine consultation who met the eligibility criteria were invited to participate by their attending physician during their clinic visit. Eligible participants were adults aged ≥18 years whose diagnosis of KOA was confirmed by trained physicians using clinical examination (assessing crepitus, pain, function, and stiffness) and the Kellgren–Lawrence (KL) radiographic grading system. A KL grade of ≥2 was required for inclusion, indicating the presence of definite osteophytes.

All participants were required to be able to read and understand the Kazakh language and to complete self-administered questionnaires independently. Patients with inflammatory arthritis, a history of recent knee surgery or acute knee trauma, severe comorbid conditions affecting mobility or pain perception, or cognitive impairment that could interfere with questionnaire completion were excluded to ensure that WOMAC responses reflected chronic knee osteoarthritis-related symptoms.

The sample size was determined using common recommendations for multivariate and factor analysis. Confirmatory Factor Analysis (CFA) typically requires at least 10 respondents per item. Given the 24 items in the WOMAC index, a minimum of 240 participants was needed. Our final sample included 452 patients with KOA, yielding an item-to-respondent ratio of about 1:19. This sample size exceeds the recommended threshold of N>400, which is generally considered sufficient for stable parameter estimates and adequate statistical power. Among the patients, a subgroup of 73 completed a repeat assessment to evaluate test–retest reliability. According to the quality criteria for health status questionnaires proposed by Terwee et al. [17] and the COSMIN methodological checklist [18], a sample size of at least 50 participants is regarded as adequate for acceptable methodological quality.

Additionally, 126 healthy individuals were recruited from the community and hospital staff specifically to assess known-groups validity. The inclusion of a healthy control group was designed to establish the discriminative validity of the Kazakh WOMAC by comparing symptomatic patients against an asymptomatic population. This comparison confirms that the instrument can accurately distinguish between the presence of disease-related symptoms and a state of normal joint function. Healthy status for these individuals was verified using a brief self-report screening questionnaire administered prior to the WOMAC. Participants were excluded if they reported a history of chronic knee or hip pain, a prior diagnosis of osteoarthritis or other joint diseases, or any musculoskeletal condition affecting daily functioning. This ensured that the control group was asymptomatic and provided a valid basis for the known-groups comparison.

### 2.2. Instrument and Cultural Adaptation

The WOMAC is a disease-specific, patient-reported tool developed to evaluate symptoms and functional limitations in individuals with knee and hip osteoarthritis. This instrument includes 24 items organized into three subscales: Pain (5 items; score range 0–20), Stiffness (2 items; score range 0–8), and Physical Function (17 items; score range 0–68). Items are scored using a Likert-type scale, with higher scores indicating greater symptom severity and functional impairment.

The translation and cultural adaptation followed the five-stage process established by Beaton et al. [9] and Guillemin et al. [19] for the cross-cultural adaptation of self-report measures. The original English version was translated into Kazakh by two independent bilingual translators whose native language was Kazakh. The first translator was a professional linguist with expertise in medical terminology, ensuring clinical concepts were accurately conveyed, while the second was a linguist with a Master’s degree in English Philology and no medical background, ensuring the wording remained accessible to patients. The translations were compared and differences were reconciled to create a single version. This version was then backward translated into English by an independent bilingual translator blinded to the original questionnaire.

The expert committee consisted of six members: two senior orthopedic surgeons, two methodology experts specializing in health-related measures, a linguist specializing in the Kazakh language, and the lead researcher. This committee compared the backward translated version with the original English version to ensure semantic, idiomatic, experiential, and conceptual equivalence. The committee’s primary role was to resolve discrepancies between the translations and to ensure that terms were culturally relevant and easily understood by the local population. A pre-final Kazakh version was pilot-tested in a small group of patients with KOA to assess clarity, comprehensibility, and cultural relevance. Minor wording adjustments were made based on participant feedback, resulting in the final Kazakh WOMAC version.

### 2.3. Data Collection

Participants completed the Kazakh WOMAC along with a demographic and clinical questionnaire, collecting information on age, sex, and relevant clinical characteristics. Clinical data included body mass index (BMI), duration of symptoms, affected side (unilateral or bilateral), and Kellgren–Lawrence (KL) radiographic grade. Information on current medication use and comorbidities was also recorded to provide a comprehensive clinical profile of the cohort. To assess convergent validity, all participants also completed the SF-36 questionnaire, using the previously validated Kazakh version [20]. A subset of participants completed the WOMAC a second time after a 7–14 day interval, during which no therapeutic changes were made, to evaluate test–retest reliability.

### 2.4. Psychometric Evaluation

#### 2.4.1. Reliability

We assessed internal consistency using Cronbach’s alpha for each WOMAC subscale and the total score, considering values ≥ 0.70 as acceptable [21]. Test–retest reliability was evaluated in a subset of 73 participants using the Intraclass Correlation Coefficient (ICC) with a two-way random effects model and absolute agreement definition (ICC[2,1]). ICC values were interpreted as: <0.50 = poor, 0.50–0.75 = moderate, 0.75–0.90 = good, and >0.90 = excellent reliability.

#### 2.4.2. Validity

Content validity was evaluated by a panel of seven bilingual experts familiar with knee osteoarthritis and questionnaire design. Each expert rated every WOMAC item on relevance, clarity, simplicity, and cultural appropriateness using a 4-point scale (1 = not at all, 4 = highly). Item-level content validity indices (I-CVI) and scale-level CVI (S-CVI/Ave) were calculated, and items with I-CVI < 0.78 were revised by consensus.

Construct validity was examined using Confirmatory Factor Analysis (CFA) with maximum likelihood estimation to determine whether the Kazakh version retained the original three-dimensional structure (Pain, Stiffness, Physical Function). Model fit was assessed with the Comparative Fit Index (CFI), Tucker–Lewis Index (TLI), Root Mean Square Error of Approximation (RMSEA), and Standardized Root Mean Square Residual (SRMR). Acceptable model fit was defined as CFI and TLI ≥ 0.90, RMSEA ≤ 0.08, and SRMR ≤ 0.08, with factor loadings ≥ 0.40 considered acceptable [22].

Convergent validity was evaluated by examining correlations between WOMAC subscales and corresponding SF-36 domains. WOMAC Pain was expected to correlate with the SF-36 Bodily Pain domain, while WOMAC Stiffness and Physical Function were expected to correlate with the SF-36 Physical Functioning domain. Pearson correlation coefficients (two-tailed, α = 0.05) were used, and scatterplots with jitter and fitted regression lines were generated to visually inspect convergence patterns.

Known-groups validity was evaluated by comparing WOMAC total and subscale scores between patients with knee osteoarthritis and healthy individuals. Because WOMAC scores in patients deviated from normality, Mann–Whitney U tests were used to compare groups, with significance set at α = 0.05.

#### 2.4.3. Floor and Ceiling Effects

Floor and ceiling effects were evaluated based on the proportion of participants achieving the lowest or highest possible scores, with effects considered present if >15% of participants achieved minimum or maximum scores [17].

### 2.5. Statistical Analysis

Descriptive statistics summarized the characteristics of participants and questionnaire scores. Continuous variables were reported as means ± standard deviations or medians with interquartile ranges, as appropriate. Categorical variables were reported as frequencies and percentages. We conducted all analyses using Stata version 18.0, with a two-sided *p*-value < 0.05 considered statistically significant.

## 3. Results

The patient group had a mean age of 51.4 ± 6.1 years (range 34–71) and a mean BMI of 30.7 ± 3.9 kg/m^2^ (range 20.8–44.7). The majority of patients were female (63.9%). Healthy participants were included to assess known-groups validity.

We evaluated the internal consistency reliability using Cronbach’s alpha. The total score showed satisfactory reliability (α = 0.77). Subscale analyses indicated good internal consistency for Pain (α = 0.83) and high reliability for Stiffness (α = 0.88), while the Physical Function subscale also demonstrated acceptable reliability (α = 0.78).

Test–retest reliability was assessed in a subgroup of 73 participants who completed the WOMAC questionnaire on two occasions. This subgroup was representative of the main patient sample, with no meaningful differences in mean age (51.5 vs. 51.4 years), BMI (30.3 vs. 30.7 kg/m^2^), or baseline WOMAC scores for pain (14.5 vs. 13.6), stiffness (5.0 vs. 5.1), and physical function (49.0 vs. 49.2). The Pain subscale showed good temporal stability (ICC = 0.83, 95% CI: 0.75–0.89), with a Standard Error Measurement (SEM) of 0.19 and Minimal Detectable Change (MDC_95_) of 0.53. Reliability for the Stiffness and Physical Function subscales was moderate to good, with ICCs of 0.78 (95% CI: 0.67–0.86; SEM = 0.29; MDC_95_ = 0.79) and 0.79 (95% CI: 0.69–0.87; SEM = 0.11; MDC_95_ = 0.31), respectively. These findings suggest that the Kazakh version of the WOMAC yields consistent and stable scores when administered on two occasions in patients with KOA.

During expert evaluation, most WOMAC items demonstrated strong content validity, with I-CVI values spanning 0.86–1.00, as shown in Table 1. However, three items initially received I-CVI values below the 0.78 threshold. Specifically, the term *bathtub* was modified to include both bath and shower options, acknowledging that many Kazakh households utilize showers rather than bathtubs. Additionally, the term *toilet* was refined to use a socially appropriate and unambiguous Kazakh term, and *going shopping* was adapted to include trips to traditional markets (bazars) to better reflect local physical activity patterns. After revision, all items achieved acceptable I-CVI values (≥0.78). The overall scale-level content validity index (S-CVI/Ave) was 0.96, indicating excellent agreement among experts regarding the relevance, clarity, simplicity, and cultural appropriateness of the Kazakh version of the questionnaire.

Confirmatory factor analysis confirmed that the WOMAC retains its original three-factor structure, corresponding to pain, stiffness, and physical function. All items loaded significantly onto their respective latent constructs (standardized loadings range: 0.35–1.00, *p* < 0.001). The model demonstrated excellent absolute and relative fit to the data: χ^2^(249) = 261.52, *p* = 0.280, χ^2^/df = 1.05; CFI = 0.994; TLI = 0.993; RMSEA = 0.011 (90% CI: 0.000–0.022); and SRMR = 0.037. These indices indicate that the Kazakh version of the WOMAC retains the structural validity of the original instrument. Notably, this fit was achieved without any post hoc modifications or the addition of error covariances.

**Figure 1:**
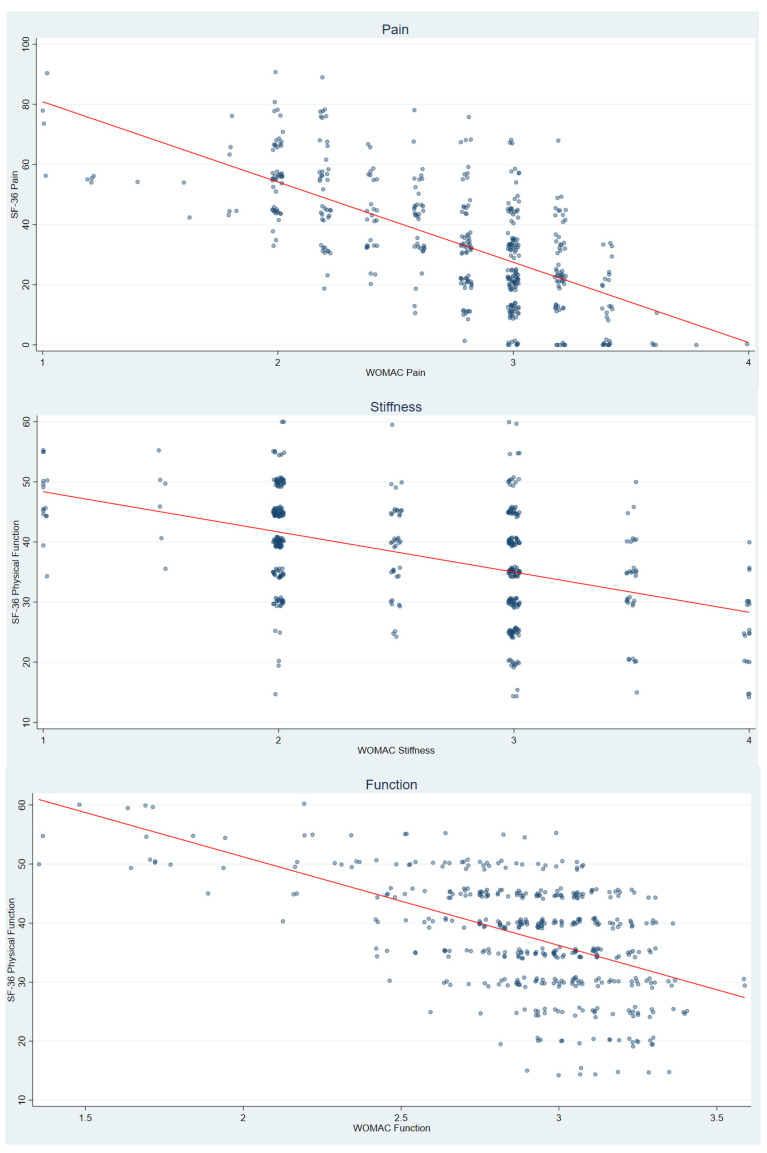
Correlations between WOMAC and SF-36 domains in KOA.

We have assessed the convergent validity by examining the associations between the WOMAC domains and conceptually corresponding domains of the SF-36 questionnaire among participants with KOA. As expected, WOMAC Pain demonstrated a moderate negative correlation with the SF-36 Bodily Pain domain (r = −0.66, *p* < 0.001), indicating good convergence between joint-specific and general pain measures. WOMAC Stiffness showed a weak-to-moderate negative correlation with SF-36 Physical Functioning (r = −0.47, *p* < 0.001), consistent with the idea that increased joint stiffness is associated with reduced overall functional capacity. WOMAC Physical Function exhibited a moderate negative correlation with SF-36 Physical Functioning (r = −0.55, *p* < 0.001). Together, these patterns provide evidence of convergent validity, with each WOMAC domain aligning most strongly with the theoretically corresponding SF-36 construct. The associated scatterplots in Figure 1 visually confirm these trends, illustrating linear relationships between matched constructs.

Known-groups validity of the Kazakh version of the WOMAC was demonstrated by statistically significant differences across all domains, with patients with KOA reporting higher scores than healthy individuals, reflecting greater symptom severity and functional limitation, as shown in Table 2. Given the non-normal distribution of scores among patients, the comparison between the groups was conducted using the Mann–Whitney U test.

Floor and ceiling effects were negligible across all WOMAC subscales in patients with KOA. No participants scored the minimum for pain, stiffness, or function. Ceiling effects were minimal: 0.22% for pain, 4.2% for stiffness, and 0.44% for function, indicating that the Kazakh WOMAC adequately captures the entire spectrum of symptom severity in this population.

## 4. Discussion

The aim of this study was to translate, culturally adapt, and examine the psychometric properties of the Kazakh version of the WOMAC in patients with KOA. Overall, the findings demonstrate that the Kazakh WOMAC is a valid and reliable tool for assessing pain, stiffness, and physical function in this population. These results support its use in both patient care and research contexts in Kazakhstan and other Kazakh-speaking contexts.

The Kazakh WOMAC demonstrated acceptable to high reliability across all domains. The psychometric performance of the Kazakh WOMAC is consistent with other validated versions in similar regional and cultural contexts. Its internal consistency (alpha = 0.77–0.88) is comparable to the Turkish (0.71–0.94) and Chinese (0.67–0.82) versions, although slightly lower than the Arabic (0.89–0.91) and Korean (0.81–0.96) versions [11,12,13,14]. Similarly, the Kazakh test–retest reliability (ICC = 0.78–0.83) aligns with the Korean version (0.79–0.89) and exceeds the acceptable threshold of 0.70 reported for the Chinese version [11,12]. These findings confirm that the Kazakh WOMAC provides stable and reliable measurements of knee osteoarthritis symptoms, consistent with international validation standards.

The variations in reliability and validity across different cultural versions of the WOMAC may stem from linguistic and lifestyle factors. For example, some languages may have more precise terms for stiffness or types of pain. This can reduce confusion for patients and lead to higher internal consistency scores in those specific versions.

Cultural differences in daily activities may also play a role. In our study, we adapted items like *using a bathtub* to include *showers* and *going shopping* to include *bazars*. These changes were necessary because a patient’s difficulty with a task might be due to an unfamiliar environment rather than their physical condition. Studies have noted that literal translations can fail if they do not account for these local habits [9]. Finally, how different cultures express or report pain can also influence the overall distribution of scores and their correlation with other health measures.

Test–retest results indicate that the instrument provides stable scores over time when no clinical change is expected, suggesting suitability for both clinical monitoring and research use.

Similarly, strong evidence of validity was observed across multiple domains. Expert review confirmed that the translated items are relevant, clear, and culturally appropriate, highlighting the importance of contextual adaptation beyond literal translation. The need for small wording adjustments in a limited number of items underscores the importance of cultural and linguistic adaptation rather than direct translation.

These challenges mirror findings from other international adaptations. The Chinese version was finalized only after resolving translation disagreements through a consensus-based approach and patient feedback. Similarly, researchers for the Arabic version modified terms like *walking on a flat surface* and *putting on socks* to achieve better linguistic fit while preserving the original intent [12,14]. These commonalities suggest that several WOMAC items require contextualization rather than literal translation to maintain psychometric equivalence across global populations.

### 4.1. Clinical and Research Implications

The availability of a validated Kazakh version of the WOMAC addresses an important gap in patient-centered assessment tools for osteoarthritis in Central Asia. Its demonstrated reliability and validity support its use for routine clinical assessment, monitoring disease progression, and evaluating treatment outcomes. In research settings, the Kazakh WOMAC enables inclusion of Kazakh-speaking populations in national and international studies and facilitates comparability of findings across countries and cultural contexts.

### 4.2. Limitations and Future Directions

Several limitations need to be noted. First, the study employed a cross-sectional design, which precludes assessment of responsiveness to treatment-related changes and disease progression over time. Consequently, the tool’s sensitivity to detect improvements following medical intervention, such as post-surgery or physiotherapy, remains to be evaluated in future longitudinal studies to confirm its responsiveness. Second, although convergent validity was assessed using the SF-36, additional comparisons with other knee-specific measures could further strengthen validity evidence.

This study was conducted at a single specialized orthopedic center, which may introduce selection bias. Patients treated in tertiary centers often have more advanced diseases and greater symptom severity than those seen in primary care. Although our sample included a range of Kellgren–Lawrence grades, the results may not fully represent patients with very early-stage osteoarthritis in community settings.

Future research should also explore measurement invariance across subgroups such as sex and age, as well as potential differences between urban and rural populations. Evaluation of electronic or interviewer-administered versions may further expand the instrument’s applicability in diverse clinical and research settings.

## 5. Conclusions

In conclusion, the Kazakh version of the WOMAC demonstrates robust validity and reliability in KOA patients. The instrument retains the original three-domain structure, shows strong content and construct validity, and effectively discriminates between patients and healthy individuals without significant floor or ceiling effects. The results indicate that the use of the Kazakh WOMAC is a culturally appropriate patient-reported outcome measure for assessing KOA in Kazakh-speaking populations.

## Figures and Tables

**Table 1:** Item- and scale-level content validity indices for the Kazakh version of the WOMAC.

Domain	Range of I-CVI	S-CVI/Ave
Pain	0.86–1.00	0.98
Stiffness	0.86–1.00	0.96
Physical function	0.86–1.00	0.96
Overall	0.86–1.00	0.96

Note. I-CVI = Item-level Content Validity Index; S-CVI/Ave = Scale-level Content Validity Index (average method). Values ≥ 0.78 indicate acceptable content validity; values ≥ 0.90 indicate excellent agreement among experts.

**Table 2:** Comparison of WOMAC domain and total scores between patients with KOA and healthy participants.

WOMAC Domain	KOA Patients (Median, IQR)	Healthy Participants (Median, IQR)	Test	*p*-Value
Pain	14 (12–15)	4 (2–5)	Mann–Whitney U	<0.001
Stiffness	5 (4–6)	2 (1–3)	Mann–Whitney U	<0.001
Physical function	50 (47–53)	26 (22–28)	Mann–Whitney U	<0.001
Total score	69 (66–72)	31 (28–35)	Mann–Whitney U	<0.001

## Data Availability

The data supporting the findings of this study, including the dataset and analysis scripts, are publicly available in Figshare at https://doi.org/10.6084/m9.figshare.31072153 accessed on 20 January 2026. The Kazakh version of the WOMAC questionnaire is available from the corresponding author upon reasonable request.

## Abbreviations

The following abbreviations are used in this manuscript:

OA	Osteoarthritis
GBD	Global Burden of Disease
KOA	Knee osteoarthritis
WOMAC	Western Ontario and McMaster Universities Osteoarthritis Index
SF-36	36-Item Short Form Health Survey
ICC	Intraclass Correlation Coefficient
I-CVI	Item-level Content Validity Index
S-CVI/Ave	Scale-level Content Validity Index (average method)
CFA	Confirmatory Factor Analysis
CFI	Comparative Fit Index
TLI	Tucker–Lewis Index
RMSEA	Root Mean Square Error of Approximation
SRMR	Standardized Root Mean Square Residual
BMI	Body Mass Index
IQR	Interquartile Range
CI	Confidence Interval

## References

[1] World Health Organization (2023). Osteoarthritis fact sheet. Global Epidemiology, Risk Factors Including Ageing and Obesity.

[2] Long H., Liu Q., Yin H., Wang K., Diao N., Zhang Y., Lin J., Guo A. (2022). Prevalence trends of site-specific osteoarthritis from 1990 to 2019: Findings from the Global Burden of Disease Study 2019. Arthritis Rheumatol..

[3] Xie X., Zhang K., Li Y., Li Y., Li X., Lin Y., Huang L., Tian G. (2025). Global, regional, and national burden of osteoarthritis from 1990 to 2021 and projections to 2035: A cross-sectional study for the Global Burden of Disease Study 2021. PLoS ONE.

[4] Bellamy N., Buchanan W.W., Goldsmith C.H., Campbell J., Stitt L.W. (1988). Validation study of WOMAC: A health status instrument for measuring clinically important patient-relevant outcomes in osteoarthritis of the hip or knee. J. Rheumatol..

[5] da Silva Júnior J.E.F., Dibai-Filho A.V., Santos I.S., Protázio J.B., Júnior J.D.A., de Oliveira D.D., Dos Santos P.G., Fidelis-de-Paula-Gomes C.A. (2023). Measurement properties of the short version of the Western Ontario and McMaster Universities Arthritis Index (WOMAC) for individuals with knee osteoarthritis. BMC Musculoskelet. Disord..

[6] Lundgren-Nilsson Å., Dencker A., Palstam A., Persson G., Horton M.C., Escorpizo R., Küçükdeveci A.A., Kutlay S., Elhan A.H., Stucki G. (2018). Patient-reported outcome measures in osteoarthritis: A systematic search and review of their use and psychometric properties. RMD Open.

[7] Adriani M., Becker R., Milano G., Lachowski K., Prill R. (2023). High variation among clinical studies in the assessment of physical function after knee replacement: A systematic review. Knee Surg. Sports Traumatol. Arthrosc..

[8] Zhao Y., Zhang Y., Liu K., Chai Y., Yan H., Lin F., Zhan H., Zheng Y., Yuan W. (2025). Test the reliability and comparability of the paper and electronic versions of the Western Ontario and McMaster University Osteoarthritis Index in Chinese: A randomized cross-sectional study. Clin. Rheumatol..

[9] Beaton D.E., Bombardier C., Guillemin F., Ferraz M.B. (2000). Guidelines for the process of cross-cultural adaptation of self-report measures. Spine.

[10] Zhang Y., Ren J., Zang Y., Guo W., Disantis A., Martin R.L. (2023). Cross-culturally adapted versions of patient-reported outcome measures for the lower extremity. Int. J. Sports Phys. Ther..

[11] Bae S.C., Lee H.S., Yun H.R., Kim T.H., Yoo D.H., Kim S.Y. (2001). Cross-cultural adaptation and validation of Korean Western Ontario and McMaster Universities (WOMAC) and Lequesne osteoarthritis indices for clinical research. Osteoarthr. Cartil..

[12] Xie F., Li S.C., Goeree R., Tarride J.E., O’Reilly D., Lo N.N., Yeo S.J., Yang K.Y., Thumboo J. (2008). Validation of Chinese Western Ontario and McMaster Universities Osteoarthritis Index (WOMAC) in patients scheduled for total knee replacement. Qual. Life Res..

[13] Tüzün E.H., Eker L., Aytar A., Daşkapan A., Bayramoğlu M. (2005). Acceptability, reliability, validity and responsiveness of the Turkish version of WOMAC osteoarthritis index. Osteoarthr. Cartil..

[14] Alghadir A., Anwer S., Iqbal Z.A., Alsanawi H.A. (2016). Cross-cultural adaptation, reliability and validity of the Arabic version of the reduced Western Ontario and McMaster Universities Osteoarthritis Index in patients with knee osteoarthritis. Disabil. Rehabil..

[15] Faik A., Benbouazza K., Amine B., Maaroufi H., Bahiri R., Lazrak N., Aboukal R., Hajjaj-Hassouni N. (2008). Translation and validation of Moroccan Western Ontario and McMaster Universities (WOMAC) osteoarthritis index in knee osteoarthritis. Rheumatol. Int..

[16] Akhmetov A., Khaumet Y., Moldaliyev I., Seksenbayev A., Oshibayeva A., Kyrykbayeva S., Nuskabayeva G., Ibragim A. (2026). Cultural practices and knee osteoarthritis in Central Asia: A case-control study on risk and protective factors. Future Sci. OA.

[17] Terwee C.B., Bot S.D., de Boer M.R., van der Windt D.A., Knol D.L., Dekker J., Bouter L.M., de Vet H.C. (2007). Quality criteria were proposed for measurement properties of health status questionnaires. J. Clin. Epidemiol..

[18] Mokkink L.B., Terwee C.B., Patrick D.L., Alonso J., Straten S.M., Knol D.L., Bouter L.M., de Vet H.C. (2010). The COSMIN checklist for assessing the methodological quality of studies on measurement properties of health status measurement instruments: An international Delphi study. Qual. Life Res..

[19] Guillemin F., Bombardier C., Beaton D. (1993). Cross-cultural adaptation of health-related quality of life measures: Literature review and proposed guidelines. J. Clin. Epidemiol..

[20] Saruarov Y., Nuskabayeva G., Gencer M.Z., Shalkharova Z., Idrissov K. (2024). Analysis of the Short Form-36 Questionnaire and Approbation of Proposals for Adaptation to the Kazakh Language. Asian J. Soc. Health Behav..

[21] Nunnally J.C., Bernstein I.H. (1994). Psychometric Theory.

[22] Hu L.T., Bentler P.M. (1999). Cutoff criteria for fit indexes in covariance structure analysis: Conventional criteria versus new alternatives. Struct. Equ. Model..

